# Screening and Functional Analyses of Novel Cecropins from Insect Transcriptome

**DOI:** 10.3390/insects14100794

**Published:** 2023-09-29

**Authors:** Lizhen Guo, Min Tang, Shiqi Luo, Xin Zhou

**Affiliations:** 1 Department of Entomology, College of Plant Protection, China Agricultural University, Beijing 100193, China; glz0127@cau.edu.cn (L.G.); min.tang@xjtlu.edu.cn (M.T.); 2 Sanya Institute of China Agricultural University, Sanya 572000, China; 3 Department of Biological Sciences, Xi’an Jiaotong-Liverpool University, Suzhou 215123, China

**Keywords:** Cecropin, antimicrobial peptide, insect, transcriptomics, genomics, 1KITE

## Abstract

**Simple Summary:**

Antimicrobial peptides (AMPs) are widely present in organisms, exhibiting broad-spectrum antimicrobial activity and rarely developing resistance. Currently, research on AMPs in many species is still limited, and they have great potential for exploration. In this study, a class of AMPs called Cecropin was identified from insect transcriptome using bioinformatics. Novel Cecropin genes were discovered in several insects and three of them were selected for experimental validation. These peptides possess antimicrobial activity against Gram-negative bacteria, exhibit a structure and mechanism of action similar to known Cecropins, and are non-toxic to mammalian cells. This study provides a reliable analytical method in the search for AMPs, which hold promising application prospects in diverse fields such as medicine, food, and beyond.

**Abstract:**

Antibiotic resistance is a significant and growing threat to global public health. However, antimicrobial peptides (AMPs) have shown promise as they exhibit a broad spectrum of antibacterial activities with low potential for resistance development. Insects, which inhabit a wide range of environments and are incredibly diverse, remain largely unexplored as a source of novel AMPs. To address this, we conducted a screening of the representative transcriptomes from the 1000 Insect Transcriptome Evolution (1KITE) dataset, focusing on the homologous reference genes of Cecropins, the first identified AMPs in insects known for its high efficiency. Our analysis identified 108 Cecropin genes from 105 insect transcriptomes, covering all major hexapod lineages. We validated the gene sequences and synthesized mature peptides for three identified Cecropin genes. Through minimal inhibition concentration and agar diffusion assays, we confirmed that these peptides exhibited antimicrobial activities against Gram-negative bacteria. Similar to the known Cecropin, the three Cecropins adopted an alpha-helical conformation in membrane-like environments, efficiently disrupting bacterial membranes through permeabilization. Importantly, none of the three Cecropins demonstrated cytotoxicity in erythrocyte hemolysis tests, suggesting their safety in real-world applications. Overall, this newly developed methodology provides a high-throughput bioinformatic pipeline for the discovery of AMP, taking advantage of the expanding genomic resources available for diverse organisms.

## 1. Introduction

The misuse of antibiotics on a global scale has resulted in a rise in drug-resistant microbes and the rapid spread of anti-drug genes [1,2,3,4]. Alternatively, antimicrobial peptides (AMPs) play a vital role in the innate immune response against pathogens and are present in organisms across all six kingdoms [5,6]. Most AMPs are short peptides that are cationic and amphipathic in nature. They have the ability to bind to the cytoplasmic membrane of target microbes, disrupting their transmembrane electrochemical gradients [7]. Additionally, AMPs play a role in inhibiting peptide synthesis, as well as promoting the synthesis of harmful reactive oxygen [8]. AMPs have demonstrated great potential in medical applications due to their wide range of antimicrobial activity, which includes the ability to target Gram-positive and Gram-negative bacteria, virus, fungi, and parasites [9,10,11]. Furthermore, their ability to evade resistance development makes them highly promising candidates [12,13].

The public Antimicrobial Peptide Database (APD) has documented over 3569 AMPs with proven antimicrobial activities across all six kingdoms [14]. However, despite being the most abundant animals on Earth, insects remain largely unexplored and offer a wealth of resources for discovering novel AMPs. Surprisingly, the APD only reports 367 insect AMPs (statistics as of June 2023), which is a significant under-representation considering the vast diversity within the insect group. Insects exhibit a wide range of AMPs in terms of both number and type. For example, the American cockroach *Periplaneta americana* is predicted to have 86 AMPs [15], while more than 50 AMPs have been identified in the ladybird *Harmonia axyridis* [16]. Conversely, none of the known AMPs have been found in the pea aphid *Acyrthosiphon pisum* [17]. Furthermore, insect AMPs display diverse structural features and are classified into three major families: peptides with alpha-helical structures lacking cysteine, peptides with beta-sheet structures, and peptides containing specific amino acid residues [10,18]. Given the high species richness, long evolutionary history, diverse habitats, and complex relationships with pathogens/symbionts in insects, it is anticipated that the insect AMPs’ repertoire holds great promise for discovering abundant novel AMPs with high efficiencies.

Cecropin, the first insect AMP, was initially discovered in the giant silkmoths *Hyalophora cecropia* [19,20], and later also identified in Lepidoptera, Diptera, and Coleoptera [10,21,22]. It exhibits potent antimicrobial activity against a wide spectrum of bacteria, including Gram-negative and some Gram-positive bacteria, as well as fungi [23,24,25]. While it is known that Cecropin likely originated after Hymenoptera diverged from the rest of Holometabola [18], the evolutionary history and function diversity of insect Cecropin have yet to be fully explored.

The identification of insect AMPs typically involves isolating peptides from pathogen-infected insects, validating their effectiveness through experiments, identifying differentially expressed genes in infected and normal insects, and cloning specific cDNAs based on homologous AMP references [26,27,28,29]. These initial studies serve as foundational references for insect AMPs, against which the vast amount of recently derived genomic data can be compared and used to discover new AMP candidates. In this study, we conducted a screening of 105 representative transcriptomes from 1000 Insect Transcriptome Evolution (1KITE) data, covering all major insect lineages, in search of homologous Cecropin sequences. This analysis led to the identification of 108 novel Cecropin sequences. To further investigate their potential, we synthesized three representative novel Cecropin peptides from the moth, flea, and scorpionfly. These newly discovered Cecropins exhibited significant antimicrobial activities against Gram-negative bacteria while demonstrating low toxicity to mammal cells. Our findings emphasize the significant potential of insect transcriptome as a valuable resource in identifying functional AMPs. This opens up new possibilities for harnessing the rapidly accumulating genomic resources available for various organisms. 

## 2. Materials and Methods

### 2.1. Screening Cecropin Homologs in 1KITE Dataset and Phylogenetic Analysis of Predicted Cecropin

The process of screening Cecropin homologs in 1KITE dataset is illustrated in Figure S1. A total of 248 known amino acid sequences of insect Cecropin (Table S1) were collected as reference sequences from the Antimicrobial Peptides Database (APD3) [14], NCBI, and the Collection of Anti-Microbial Peptides (CAMP) [30]. To eliminate redundancy, these sequences were clustered with CD-HIT (version 4.7, with an identity threshold of 95%) [31]. Homology searches for Cecropin in the representative 1KITE dataset (https://1kite.cngb.org (accessed on 18 August 2020)) [32] were performed using BLASTP (e-value 1 × 10^−5^) and HMMER (version 3.0, with default parameters) [33], employing all possible open reading frames (ORFs) translated from the transcriptome as queries. The identified peptides were aligned with the NR database, and those displaying the best match with genes other than Cecropin were considered as false positive results and subsequently excluded. 

We applied a more stringent redundancy elimination procedure to the reference sequences using CD-HIT (version 4.7, with an identity threshold of 75%) [31]. This led to a final cluster of 60 representative sequences. These filtered and selected references were used to construct the phylogenetic tree of Cecropin. Multiple alignments of the Cecropin gene were performed using the Muscle algorithm in the MEGA 11 software package [34] with default arguments and then manually refined. Maximum likelihood (ML) phylogenetic trees were generated with RAxML-HPC v.8 on XSEDE in CIPRES Science Gateway, using 1000 rapid bootstrap replicates. The PROTGAMMA model was selected for ML. To enhance the visualization of the phylogenetic tree, the iTOL website (https://itol.embl.de/ (accessed on 23 July 2023)) was utilized.

### 2.2. cDNA Cloning of Representative Cecropin Homologs

Total RNA was extracted from *Phyllodes eyndhovii* from Lepidoptera, *Panorpa vulgaris* from Mecoptera, and *Tunga penetrans* from Siphonaptera as part of the 1KITE project. The RNA (2–5 µg) was reverse transcribed into cDNA using Oligo (dT)_15_ Primer and M-MLV reverse transcriptase (Promega, WI, USA) in a 50 µL reaction volume, with the reverse transcription mixture incubated at 42 °C for 1 h. The primers are listed in Table 1. The PCR mixture (25 µL) consisted of 5 µL of cDNA template, 0.2 µM primers, 200 µM dNTP mix, and 2.5 U of TaKaRa Ex Taq polymerase (Takara, Shiga, Japan). The amplification conditions were as follows: initial denaturation at 94 °C for 5 min, followed by 35 cycles of denaturation at 94 °C for 30 s, annealing at 50 °C for 30 s, and extension at 72 °C for 30 s, with a final extension at 72 °C for 10 min. All PCR products were sequenced using Sanger sequencing (Ruibiotech, Beijing, China).

### 2.3. Sequence Analysis of Predicted Cecropin

The CDS sequences of the identified *cecropin* genes were determined using ORFfinder (https://www.ncbi.nlm.nih.gov/orffinder/ (accessed on 10 September 2020)). The molecular mass (MW), isoelectric point (pI), and grand average of hydropathicity (GRAVY) of the mature peptides were calculated using ProtParam (http://www.expasy.ch/tools/protparam.html (accessed on 10 September 2020)). The presence of signal peptide was predicted using SignalP4.0 (http://www.cbs.dtu.dk/services/SignalP/ (accessed on 10 September 2020)). The hydrophobicity and hydrophilicity of mature peptides were computed with ProtScale (https://web.expasy.org/protscale/ (accessed on 10 September 2020)). The helical wheel projection of each peptide was also performed using http://rzlab.ucr.edu/scripts/wheel/wheel.cgi. (accessed on 10 September 2020). SMART (http://smart.embl-heidelberg.de/ (accessed on 10 September 2020)) was used to predict the membrane-spanning regions. The three-dimensional (3D) structure of each peptide was generated using Swiss-model (https://www.swissmodel.expasy.org (accessed on 10 September 2020)). 

### 2.4. Peptide Synthesis of Representative Cecropin Homologs

Three mature peptides, namely Peyn-cec (from *P. eyndhovii*, Lepidoptera), Pvul-cec (from *P. vulgaris*, Mecoptera), and Tpen-cec (from *T. penetrans*, Siphonaptera), along with Cecropin-B identified from the giant silkworm *H. cecropia* (Hcec-cecB, used as a positive control), were synthesized using the solid-phase FMOC method by Zhejiang Ontores Biotechnologies Company (Hangzhou, China). These synthesized peptides were purified using high-performance liquid chromatography (HPLC) to achieve a purity level higher than 90%, and were further confirmed by mass spectrometer (MS) analysis. The peptides were dissolved in a 10 nM phosphate buffer solution (PBS, pH = 7.4) and sterilized using syringe filters with a pore size of 0.22 µm.

### 2.5. Secondary Structure Analysis of Cecropin with Circular Dichroism (CD)

The secondary structure of the synthesized peptides was determined by conducting CD spectroscopic analysis at room temperature using a Bio-Logic MOS 450 CD spectrometer (Bio-Logic, Grenoble, France). The peptide samples were dissolved in three different solutions with a final concentration of 0.2 mg/mL: 10 mM PBS (pH = 7.4), trifluorethanol (TFE, 50%, *v*/*v*), and 20 mM SDS. The spectra were obtained by averaging three scans recorded from 190 nm to 250 nm at a scanning speed of 30 nm/min.

### 2.6. Antimicrobial Assays

The antimicrobial activity of the synthesized peptides was evaluated against two Gram-negative bacteria, *Escherichia coli* (ATCC 25922) and *Pseudomonas aeruginosa* (CGMCC 1.10712); as well as two Gram-positive bacteria, *Staphylococcus aureus* (ATCC 6538) and *Micrococcus luteus* (ATCC9341). The bacteria were cultured in Mueller–Hinton (MH) broth at 37 °C until the logarithmic growth phase was reached. After centrifugation, the microorganisms were re-suspended in 0.9% NaCl solution to achieve a concentration of 10^6^ colony forming units per milliliter (CFU/mL). 

For the Minimum Inhibitory Concentration (MIC) assay, 100 µL of the microorganisms were mixed with 100 µL of serial dilutions of synthesized peptides, resulting in final concentrations of 0.5, 1, 2, 4, 8, 16, 32, 64 µg/mL (0.1, 0.2, 0.4, 0.8, 1.6, 3.2, 6.4, 12.8 μg). The negative control contained only PBS. The mixture was incubated for 18 h and then transferred to 96-well plates. The absorbance values at 600 nm were recorded using the ELX-800 Absorbance Microplate Readers (Bio-Tek, Winooski, VT, USA). For the Cecropins that did not show effects in the MIC test under these AMP concentrations, 128 μg/mL AMPs were also used in the MIC test. The MIC assay was conducted in triplicates and repeated for three times. 

Additionally, the agar well diffusion assay was performed on the four aforementioned bacteria. The bacteria solution was spread on Mueller–Hinton agar plates, and then 20 µg of the synthesized peptides was added to a 6 mm well on the agar plates. After incubation at 37 °C for 18 h, the inhibition zone was measured. Ampicillin (20 µg) and PBS were used as positive and negative controls, respectively. The agar well diffusion assay was performed in triplicates.

### 2.7. Permeability of Bacteria Membrane

As the Cecropin family exerts its action through bacterial membrane lysis [35], propidium iodide (PI) staining was applied to *E. coli* ATCC25922 to determine if the bacterial membrane was permeabilized by the synthesized peptides. Upon penetration of cell membranes by the peptides, bacterial nuclei were released and subsequently stained with PI, allowing for visualization under a fluorescence microscope. Bacteria in the logarithmic phase were centrifuged at 2000 rpm for 5 min. The resulting pellets were washed three times with PBS and then diluted to a concentration of 10^8^ CFU/mL. The bacteria solution was mixed with synthesized peptides at a final concentration 4 µg/mL and incubated at 37 °C for 1 h. PI staining was carried out using the Apoptosis and Necrosis Assay Kit (Beyotime Biotechnology, Shanghai, China). The condensed or fragmented nuclei of apoptotic cells were observed using a fluorescence microscopy (Zeiss, Jena, Germany).

### 2.8. Hemolytic Assay

Given that AMPs are often used in food or medical products, we conducted a hemolytic assay to assess the potential toxicity of the synthesized Cecropins to living cells. Fresh, sterilized, defibrinated sheep blood was used in this assay. First, 4 mL of blood was centrifuged at 1000 rpm for 10 min at 4 °C, and the resulting pellets were washed three times with PBS (centrifuged at 1000 rpm for 10 min at 4 °C) until the supernatant was clear. The blood cells were diluted with PBS to a final concentration of 4% (*v*/*v*). 

Next, different concentrations of the synthesized peptides (200 µL, final concentrations of 1, 5, 20, 50 µg/mL) were mixed with 200 µL of blood cells and incubated at 37 °C for 1 h. The mixture was then centrifuged at 1000 rpm for 10 min, and the supernatants were transferred to 96-well plates. The absorbance values of the supernatants were measured at 540 nm using ELX-800 Absorbance Microplate Readers (Bio-Tek, VT, USA). For the positive and negative controls, the blood cells were incubated in 0.1% Triton-X (100% hemolysis) and PBS (0% hemolysis), respectively. The hemolytic percentage was calculated using the formula: (Absorbance_peptide_ − Absorbance_PBS_)/(Absorbance_Triton X-100_ − Absorbance_PBS_) × 100. Each peptide was tested in five replicates and the experiment was repeated three times. 

### 2.9. Statistical Analysis

The data are presented as mean ± SD. The statistical analyses were performed using two-way ANOVA test or multiple t test. Differences were considered significant when the *p* value < 0.05.

## 3. Results

### 3.1. Identification of Cecropin Genes from 1KITE

We identified a total of 108 novel putative Cecropin-like genes from 29 out of 105 1KITE transcriptomes (Table S2) using BLAST and HMMER. Among these, 77 sequences were identified via both screening methods, while an additional three sequences were exclusively detected through the HMMER screening method. The Blast screening method exhibited greater efficiency compared to the HMMER method, which specifically detected 28 homologous sequences of Cecropin. In addition to the previously reported Cecropin genes from Lepidoptera, Diptera, and Coleoptera [10,21,22], we also identified novel Cecropin genes in the orders Trichoptera, Neuroptera, Megaloptera, Mecoptera, and Siphonaptera. Multiple *cecropin* genes were found in 22 species, particularly in the sister lineages of Lepidoptera and Trichoptera. For example, we identified a total of 21 Cecropin genes in the orange swift moth *Triodia sylvina*, while only seven were found in the caddisfly *Platycentropus radiates*. The extensive diversity of *cecropin* genes observed in Lepidoptera and Trichoptera species suggests a significant role for Cecropin in these two groups. 

**Figure 1:**
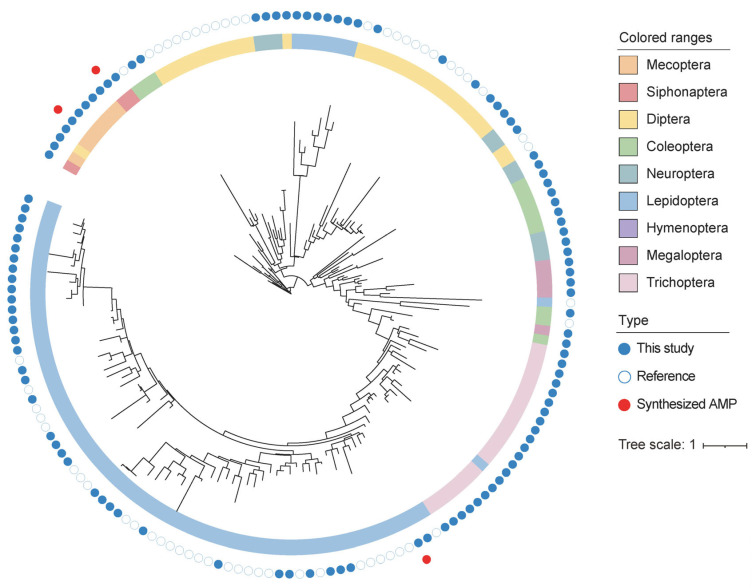
The Maximum Likelihood tree of identified novel Cecropins. The sequences obtained from varied insect orders are shaded in different colors. The sequences identified in this study are marked in red. The genes for antimicrobial activity assays, namely Peyn-cec, Pvul-cec, and Tpen-cec, are highlighted in bold.

To understand the evolutionary history of *cecropin* in insects, we constructed a phylogenetic tree using all insect Cecropin sequences from NCBI (with redundancy removed by CD-HIT at 75% identity) and the newly identified Cecropin. The predicted Cecropin genes within the same insect order exhibited a higher degree of phylogenetic relatedness to each other (Figure 1). The Cecropin genes from Lepidoptera and Trichoptera showed a higher degree of similarity and were closely related. In Diptera, the Cecropin genes mainly identified from Cyclorrhapha are clustered together, while those identified from Culicidae are also clustered together. The number of *cecropin* genes from other insect orders was small, and their relationship did not match the species phylogeny. For example, Cecropin genes from Neuroptera were closer to those from Diptera rather than Megaloptera, suggesting a potentially complex evolutionary history of Cecropin.

### 3.2. Cecropin Sequence Validation and Characteristics

**Figure 2:**
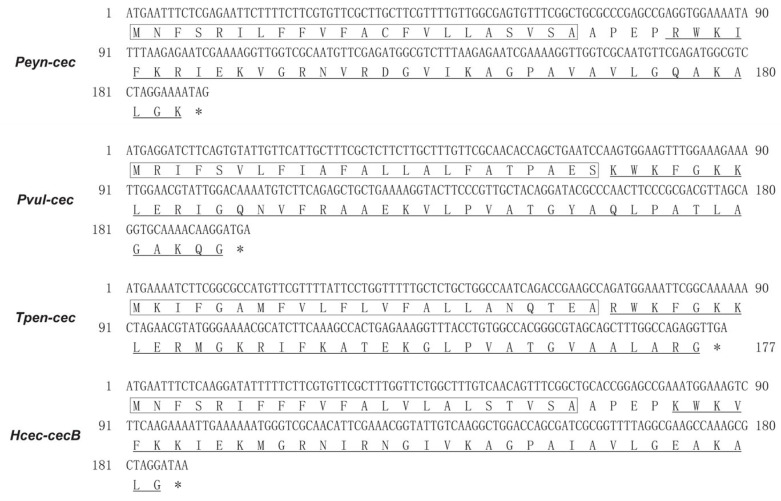
Nucleic and amino acid sequences encoding Peyn-cec, Pvul-cec, Tpen-cec, and Hcec-cecB. Nucleotide positions are indicated by numbers. Putative signal peptides are enclosed in boxes. The synthesized mature peptides are indicated by underscores, and the stop codon is indicated by an asterisk symbol (*).

To assess the antimicrobial efficiency and toxicity of the identified putative Cecropin-like peptides, we selected three novel Cecropin-like genes from different orders of insects: *Phyllodes eyndhovii* (order Lepidoptera, abbreviated as Peyn-cec), *Panorpa vulgaris* (order Mecoptera, abbreviated as Pvul-cec), and *Tunga penetrans* (order Siphonaptera, abbreviated as *Tpen-cec*). It is worth noting that *cecropin* was not identified previously, nor was the antimicrobial activity validated from the Siphonaptera and Mecoptera. Initially, the identified sequences were validated through RT-PCR analysis. The ORFs of Peyn-cec, Pvul-cec, and Tpen-cec are 192, 198, and 177 bp, respectively, with encoding proteins consisting of 63, 65, and 58 amino acids (Figure 2). Peyn-cec exhibited 90% similarity to Cecropin-A2 from *Hyphantria cunea* (Lepidoptera, Genbank accession P50722.1), while Pvul-cec showed 51% identity to Cecropin from the mosquito *Anopheles darlingi* (Diptera, Genbank accession ETN64455). The best hit in the BLASTP alignment for Tpen-cec revealed a 55% identity to a Cecropin-B2-like isoform X2 from the emerald ash borer *Agrilus planipennis* (Colecoptera, GenBank accession XP_025833989). Peyn-cec, Pvul-cec, and Tpen-cec contained an N-terminal signal peptide and a transmembrane helix (indicated by red letters and black boxes in Figure 2, respectively), resembling typical precursors of known Cecropins. 

**Figure 3:**
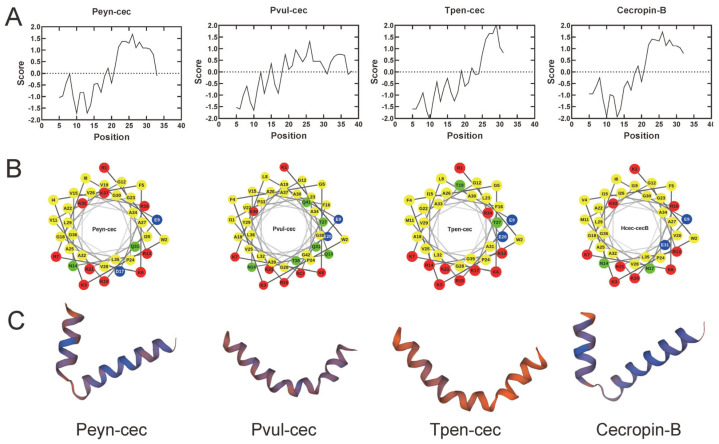
Hydrophobicity/hydrophilicity analysis and three-dimensional structure of the synthesized peptides of Peyn-cec, Pvul-cec, Tpen-cec, and Hcec-cecB. (**A**) The Kyte and Doolittle hydropathicity score. A higher score indicates higher hydrophobicity. (**B**) The helical wheel projection. Residues are numbered from the N-terminus. Circles: hydrophilic residues; diamonds: hydrophobic residues; triangles: potentially negatively charged residues; pentagons: potentially positively charged residues. Hydrophobicity is also color coded. Green: the most hydrophobic residues. Yellow: zero hydrophobicity. Red: the most hydrophilic residues. Light blue: the potentially charged residues. (**C**) Theoretical three-dimensional structure projection of peptides.

We used multiple alignments with known Cecropins to infer the mature peptide sequences of Peyn-cec, Pvul-cec, and Tpen-cec, and then synthesized these peptides. The synthesized Peyn-cec, Pvul-cec, and Tpen-cec are 37, 42, and 35 amino acids (aa) in length, with molecular weights of 4.03 kDa, 4.54 kDa, and 3.87 kDa, and isoelectric points of 11.60, 10.38, and 11.6, respectively. The hydropathicity indicators of GRAVY are −0.162, −0.279, and −0.386, and the net charges are +8, +6, and +8 for Peyn-cec, Pvul-cec, and Tpen-cec, respectively. Hydrophobicity/hydrophilicity analyses and helical wheel projections indicated that all peptides are amphipathic: the N-terminal has relatively high hydrophilicity, while the C-terminal is more hydrophobic (Figure 3A,B). This is a typical feature of known Cecropins.

**Figure 4:**
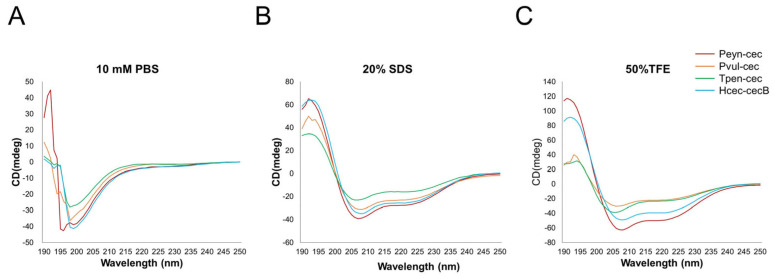
The circular dichroism (CD) spectra of peptides in different environments. Different colors represent different peptides. Red line: Peyn-cec; Orange line: Pvul-cec; Green line: Tpen-cec; Deepsky; Blue line: Hcec-cecB. The peptides were dissolved in 10 mM phosphate-buffered saline (PBS, pH 7.4) (**A**); 20 mM sodium dodecyl sulfate (SDS) (**B**); and 50% trifluoroethanol (TFE) (**C**).

The predicted secondary structure of the synthesized Peyn-cec, Pvul-cec, and Tpen-cec was mainly of an alpha-helix, similar to Hcec-cecB (Figure 3C). CD spectroscopy was used to determine their secondary structures, which showed that both the synthesized peptides and Hcec-cecB exhibited “random coil” conformations in PBS (Figure 4). In membrane-mimetic environments such as 20 mM SDS and 50% TFE solutions, Peyn-cec, Pvul-cec, and Tpen-cec displayed two minimum adsorptions at 208 and 222 nm, respectively, similar to Hcec-cecB, which is characteristic of α-helix (Figure 4).

### 3.3. Antimicrobial Efficiency and Hemolytic Activity of the Novel Cecropins

**Figure 5:**
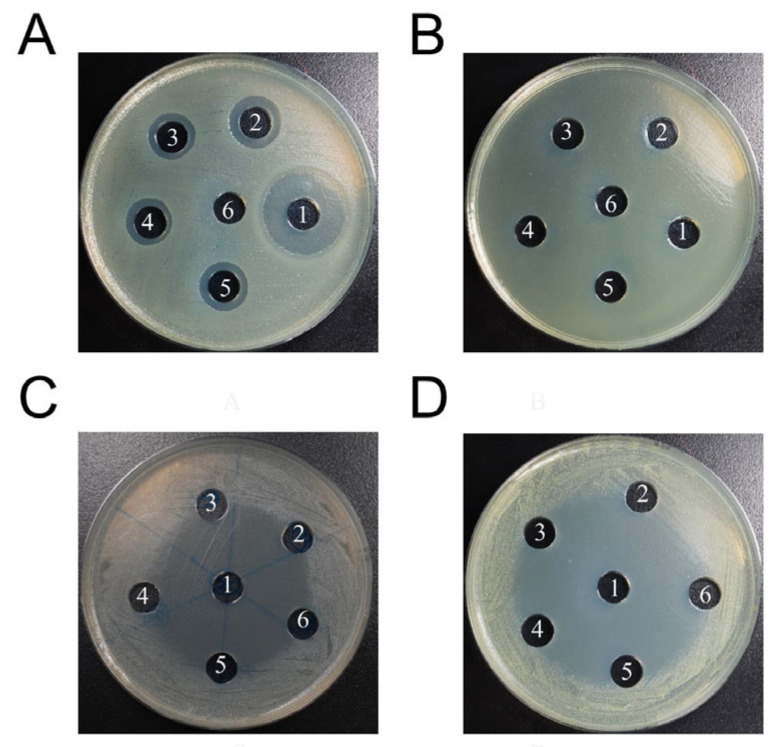
Antimicrobial activities against *E. coli* (**A**), *P. aeruginosa* (**B**), *S. aureus* (**C**), and *M. luteus* (**D**). 1: Ampicilin; 2: Peyn-cec; 3: Pvul-cec; 4: Tepn-cec; 5: Hcec-cecB; 6: PBS (negative control). A total of 20 µg of peptide solution or ampicillin was added into each hole.

The synthesized Cecropins displayed antimicrobial activity against Gram-negative bacteria, but not Gram-positive bacteria in the MIC assay with the four microorganisms analyzed (Table 2). A smaller MIC value indicated a higher antimicrobial efficiency. In general, the three newly discovered Cecropins showed similar or slightly lower efficiencies against the two Gram-negative bacteria compared with the known Hcec-cecB. In the agar well diffusion assay, none of the Cecropins exhibited noticeable effects against Gram-positive bacteria, while ampicillin demonstrated antimicrobial efficacy (Table 3, Figure 5). All tested drugs exhibited inhibitory effects on *E. coli*; however, the antibacterial peptides synthesized showed significantly lower antimicrobial effects compared to the positive control, ampicillin (multiple *t* test, *p* < 0.0001). The inhibitory effect of Pvul-cec on *E. coli* was slightly superior to that of Hcec-cecB, resulting in the largest diameter of the inhibition zone (Figure 5). However, no statistically significant difference was observed between these two AMPs (multiple t test, *p* = 0.31). The diameter of the inhibition zone for Peyn-cec and Tpen-cec was comparable but smaller than that of Hcec-cecB. Nevertheless, there were no significant differences observed among these three AMPs. It is worth noting that both Peyn-cec and Tpen-cec exhibit significantly lower antibacterial activity against *E. coli* compared to Pvul-cec (multiple *t* test, *p* = 0.020 and *p* = 0.032). Additionally, Cecropin displayed a longer duration of action compared to ampicillin. After 18 h of incubation, the bacteria started to recolonize the inhibition zone induced by ampicillin, while the boundaries of the Cecropin-induced inhibition zones remained clear. 

**Figure 6:**
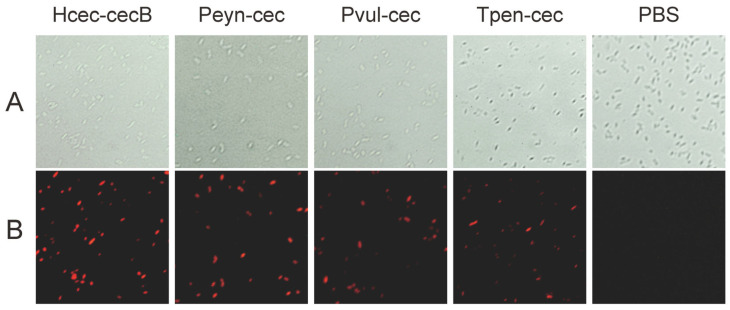
Membrane permeabilization of *E. coli* by Peyn-cec, Pvul-cec, and Hcec-cecB. *E. coli* (1 × 10^6^ CFU/mL) were incubated with 4 µg/mL of antimicrobial peptides for 1 h at 37 °C. The bacteria were stained with propidium iodide (PI) and visualized under an optical microscope (**A**) and a fluorescence microscope (**B**).

After incubation for 1 h, all Cecropin-treated bacteria displayed clear evidence of membrane damage (shown as red fluorescence in Figure 6). This suggests that membrane permeabilization is a possible mechanism for Peyn-cec, Pvul-cec, and Tpenn-cec. In summary, our results from the MIC and agar well diffusion assays indicate that Peyn-cec, Pvul-cec, and Tpen-cec have similar antimicrobial spectra to Hcec-cecB, particularly against the Gram-negative bacteria tested. 

Before clinical application, the hemolytic activities of AMPs need to be evaluated. Chemically synthesized Cecropins exhibited low hemolytic capacity against sheep red blood cells, with a maximum hemolysis rate of only 6.37 % even after treatment with Peyn-cec at 50 μg/mL for 1 hour (Table 4). Although all AMPs have low hemolytic activity, the hemolysis rate of Peyn-cec is significantly higher than the other three AMPs at concentrations of 20 µg/mL and 50 µg/mL (two-way ANOVA test, *p* < 0.001). However, no significant differences in hemolysis rates were observed among the other three AMPs.

We also compared the effect of AMP concentration on red blood cell hemolysis for each peptide. The hemolysis rate in the Hece-cecB treatment group exhibited positive correlation with peptide concentration, and significant differences were observed among different concentrations (multiple *t* test, *p* < 0.0001). In the Peyn-cec treatment group, there was no significant difference in hemolysis rates between the concentration groups of 20 µg/mL and 50 µg/mL; however, both concentrations exhibited higher hemolysis rates compared to the 5 µg/mL concentration group (multiple *t* test, *p* = 0.0032 and *p* = 0.0010, respectively). Moreover, at the concentration of 1 µg/mL, Peyn-cec exhibited lower hemolysis rates compared to the 5 µg/mL group (multiple *t* test, *p* = 0.0058). In the Pvul-cec and Tpen-cec treatment groups, there were no significant differences in hemolysis rate at different concentrations of AMPs.

## 4. Discussion

Over the past few years, AMPs derived from insects have gained significant attention as potential therapeutic agents. This is due to their broad-spectrum antimicrobial activity, low hemotoxicity, and immunomodulatory properties [12,36,37]. However, the traditional purification and cloning approach from bacteria-infected insects has made it difficult to identify AMPs from non-model or wild insects. Fortunately, with the development of next-generation sequencing, AMP identification through homologous searching has become an effective approach [15,38,39,40]. One of the earliest identified AMP is Cecropin, known for its high-efficiency in killing bacteria. In this study, we used the 1KITE data to explore the Cecropin gene from representative insect orders. We identified 108 novel *cecropin* genes, which unveils a comprehensive understanding of the distribution and evolution of Cecropin in insects. Importantly, the Cecropin genes from the five insect orders (Mecoptera, Megaloptera, Neuroptera, Siphonaptera, and Trichoptera) have been publicly known for the first time.

Cecropins represent a prominent and highly diverse family of immune effectors in insects, exhibiting remarkable potency and abundance [41]. It is hypothesized that a common ancestor of Cecropin exists among all holometabolous insects, with the exception of Hymenoptera [18]. Our research findings provide empirical evidence to support this hypothesis. By constructing a phylogenetic tree based on predicted Cecropin genes, it becomes evident that insects belonging to Diptera and Lepidoptera continue to play a prominent role in the production of Cecropin genes. In *Drosophila*, the Cecropin genes form a multigene family that is organized in tandem [42]. The high diversity of Cecropin may be attributed to the ecological niche of insects, which might encounter more pronounced microbial challenges.

The high-throughput screening of AMPs using bioinformatics methods is increasingly prevalent [43,44]. However, previous studies often focused on AMP screening using a single method, such as BLAST or HMMER [45,46,47]. There are differences in screening principles between the two methods, so combining them for AMP screening may enhance efficiency. In this study, we integrated these two approaches to screen for AMPs belonging to the Cecropin family. Our study found that BLAST was more efficient in identifying AMPs in distinct insect orders. We did not identify *cecropin* genes in all tested insect species, possibly because certain *cecropin* genes are lowly expressed in wild non-bacteria-infected insects. Although we did not extensively validate the predicted Cecropins, we validated three sequences and synthesized the mature peptides for preliminary functional analysis. All chemically synthesized AMPs exhibited typical features and modes of action of the Cecropin family [25,48]. Pvul-cec and Tpen-cec were discovered in the orders Mecoptera and Siphonaptera, respectively. It is worth noting that no Cecropin had been documented in these two insect orders before. Our results bridge this knowledge gap and suggest that other screened sequences could potentially exhibit comparable functionalities of great scientific interest.

Functional analysis plays a crucial role in the application of AMPs. Specifically, Peyn-cec, Pvul-cec, and Tpen-cec exhibit potent activity against Gram-negative bacteria while showing limited or no efficacy against Gram-positive bacteria. The anti-bacteria spectrum is similar to that of Hcec-cecB, with slightly varying suppression effects, which may be attributed to different amino acid compositions. In the case of Pvul-cec, we conducted an experiment where the last four amino acids “AKQG” were removed to examine the impact on antibacterial effects. The results demonstrated an enhancement in antibacterial activity against *P. aeruginosa* CGMCC 1.10712, with MIC changing from 32 μg/mL to 8 μg/mL (preliminary data). This finding suggests the potential of AMP engineering and design [49,50]. Importantly, the specific suppression effects on Gram-negative bacteria (such as *P. aeruginosa* in the study), which ampicillin fails to eliminate, along with low toxicity to mammalian cells, make the novel Cecropins a promising substitution or addition to conventional antibiotics.

The potential of AMPs for translational application is significant. However, harnessing their full potential will require a more sophisticated foundational understanding. The diversity of AMPs in insects is remarkably abundant, and distinct AMPs function synergistically in vivo [51,52]. Moreover, emerging evidence suggests that AMP activities in biological contexts may not be adequately captured via conventional in vitro assays [53]. Therefore, when considering the application of insect AMPs in practice, in addition to designing them through existing methods to enhance their antibacterial activity and stability, we also need to focus on the fundamental research of AMPs in insect species and incorporate the characteristics of AMP synergistic effects into consideration.

In conclusion, our study has developed an efficient and sensitive bioinformatic pipeline for the high-throughput screening of *cecropin*, which is also valuable for the exploration of other important proteins. We have successfully identified novel insect *cecropin* and validated the functions of three representative novel Cecropins. With the abundance of insect species and the increasing genomic and transcriptome data, the exploration and application of novel AMPs are expected to progress rapidly.

## Figures and Tables

**Table 1:** Primers used in cDNA cloning.

Gene	Primers in 5′-3′ Direction
*Peyn-cec*	CAGCATCCGAGAACAAAAGAACT
	TGCTTCTTAAAATATAATGAAT
*Pvul-cec*	GGTGATACCGAAAGTTCACAGC
	AATTCATCCTTGTTTTGCACCTG
*Tpen-cec*	TCGTACATCTGAAGTGAAGA
	ATGCCTAATCAACCTCTGG

**Table 2:** Minimum inhibitory concentration (MIC) of Pvul-cec, Tpen-cec, and Hcec-cecB against bacteria.

Peptide			MIC (µg/mL)	
	Peyn-cec	Pvul-cec	Tpen-cec	Hcec-cecB
Gram-positive bacteria				
*M. luteus* ATCC9341	>128.0	>128.0	>128.0	>128.0
*S. aureus* ATCC6538	>128.0	>128.0	>128.0	>128.0
Gram-negative bacteria				
*E. coli* ATCC25922	8.0	8.0	8.0	8.0
*P. aeruginosa* CGMCC 1.10712	32.0	32.0	32.0	16.0

**Table 3:** Inhibition zone of Peyn-cec, Pvul-cec, Tpen-cec, and Hcec-cecB against bacteria.

Peptide		Zone of Inhibition (mm)			
	Peyn-cec	Pvul-cec	Tpen-cec	Hcec-cecB	Ampicillin
Gram-positive bacteria					
*M. luteus* ATCC9341	-	-	-	-	59.49 ± 0.79
*S. aureus* ATCC6538	-	-	-	-	49.57 ± 1.69
Gram-negative bacteria					
*E. coli* ATCC25922	13.30 ± 0.75	15.43 ± 0.56	13.46 ± 0.37	14.30 ± 0.24	25.18 ± 1.10
*P. aeruginosa* CGMCC 1.10712	-	-	-	-	<6

**Table 4:** Hemolytic effects of Peyn-cec, Pvul-cec, Tpen-cec, and Hcec-cecB against sheep red blood cells.

Peptides	Hemolysis (%)			
	1 µg/mL	5 µg/mL	20 µg/mL	50 µg/mL
Peyn-cec	0.07 ± 0.24%	2.71 ± 0.78%	5.78 ± 0.58%	6.37 ± 0.57%
Pvul-cec	2.01 ± 0.76%	1.59 ± 1.18%	1.90 ± 0.72%	2.19 ± 0.92%
Tpen-cec	0.05 ± 0.15%	0.67 ± 0.48%	1.32 ± 1.24%	1.72 ± 1.28%
Hcec-cecB	0.46 ± 0.07%	0.97 ± 0.77%	1.25 ± 0.20%	1.75 ± 0.57%

## Data Availability

The data presented in this study are available in the Supplementary Information.

## Supplementary Materials

The following supporting information can be downloaded at: https://www.mdpi.com/article/10.3390/insects14100794/s1, Figure S1: The pipeline of Cecropin prediction; Table S1: Information of insect *cecropin* references; Table S2: Number of Cecropin homologous genes in the representative transcriptome from 1KITE.
