# Supplementary figures and images for: Screening and Functional Analyses of Novel Cecropins from Insect Transcriptome
Source: Insects. 2023 Sep 29;14(10):794. doi: 10.3390/insects14100794 (PMC10607850; doi:10.3390/insects14100794)

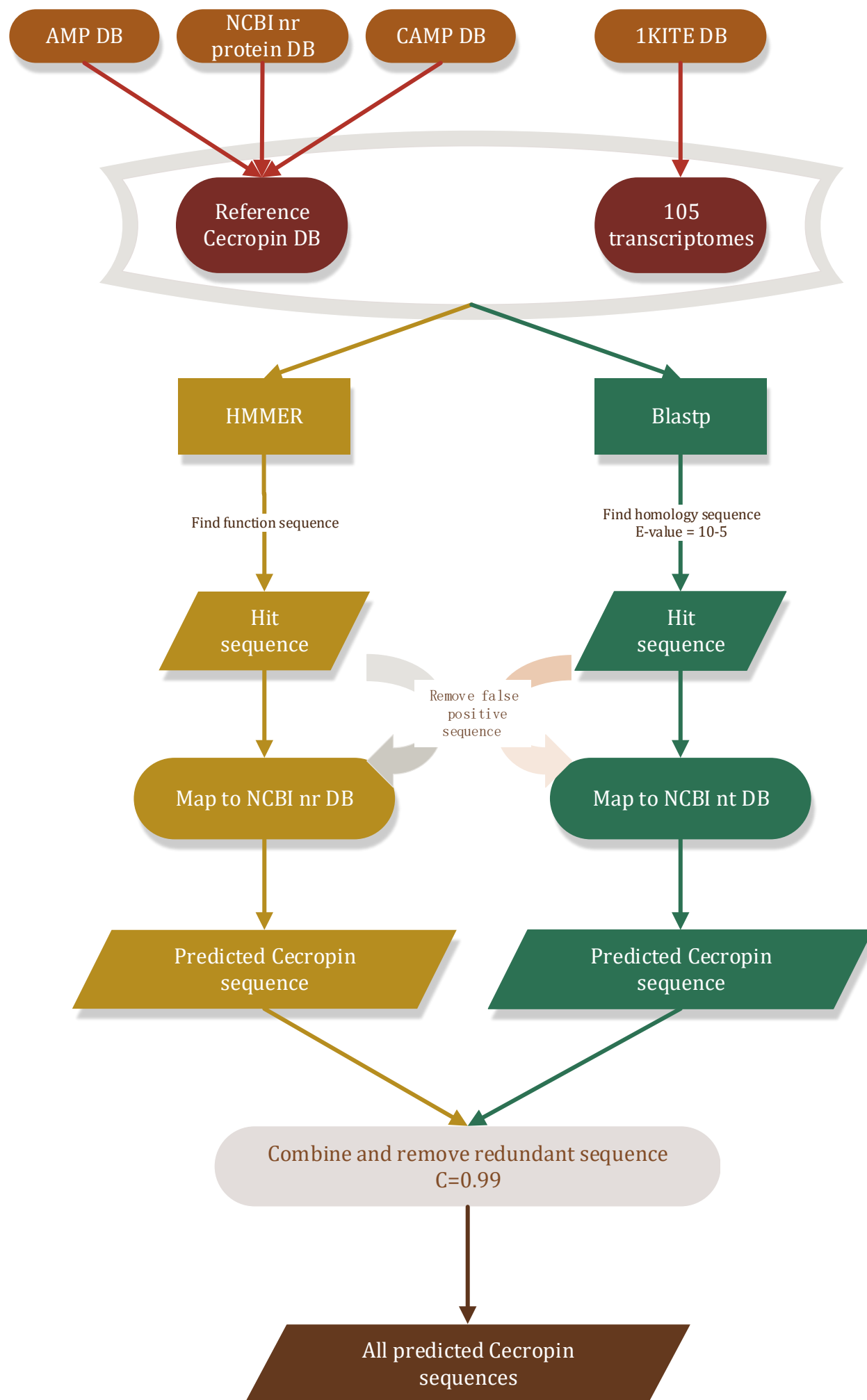

Supplement: Supplementary file 1 [file insects-14-00794-s001.zip › figureS1.pdf]
